# Remnants of Cardinal Symptoms of Parkinson's Disease, Not Dyskinesia, Are Problematic for Dyskinetic Patients Performing Activities of Daily Living

**DOI:** 10.3389/fneur.2019.00256

**Published:** 2019-03-22

**Authors:** Etienne Goubault, Hung P. Nguyen, Sarah Bogard, Pierre J. Blanchet, Erwan Bézard, Claude Vincent, Justyna Sarna, Oury Monchi, Christian Duval

**Affiliations:** ^1^Département des Sciences de l'Activité Physique, Université du Québec à Montréal, Montréal, QC, Canada; ^2^Centre de Recherche de l'Institut Universitaire de Gériatrie de Montréal, Montréal, QC, Canada; ^3^Département de Stomatologie, Faculté de Médecine Dentaire, Université de Montréal, Montréal, QC, Canada; ^4^Département de Médecine, CHU Montréal, Montréal, QC, Canada; ^5^Laboratoire de Neurophysiologie, Université de Bordeaux, Institut des Maladies Neurodégénératives, Bordeaux, France; ^6^Unité Mixte de Recherche 5293, Centre National de la Recherche Scientifique, Institut des Maladies Neurodégénératives, Bordeaux, France; ^7^Département de Réadaptation, Faculté de Médecine, Université Laval, Quebec, QC, Canada; ^8^Department of Clinical Neurosciences, Hotchkiss Brain Institute, University of Calgary, Calgary, AB, Canada

**Keywords:** Parkinson disease, levodopa-induced dyskinesia, activities of daily living, motor performance, cardinal symptoms

## Abstract

**Introduction:** The impact of levodopa-induced dyskinesia (LID) on the daily lives of patients with Parkinson's disease (PD) remains to be determined. Furthermore, evidence suggests that cardinal motor symptoms of PD may coexist with LID, but their impact on activities of daily living (ADL) relative to LID is not known. This cross-sectional study aimed at determining the effect of LID and cardinal motor symptoms of PD on ADL in patients who were experiencing peak-dose choreic-type LID.

**Method:** One hundred and twenty-one patients diagnosed with PD known to experience choreic-type LID were recruited for the study. Patients were asked to perform a set of ADL. Levels of LID, tremor, bradykinesia, and freezing of gait (FoG) were measured using 17 inertial sensors design to capture full body movements, while rigidity, and postural instability were assessed using clinical evaluations. Cognition was also assessed using the mini-mental state examination. Success criteria were set for each ADL using the time needed to perform the task and errors measured in 69 age-gender-matched healthy controls. Binary logistic regressions were used to identify symptoms influencing success or failure for each activity. Receiver operating characteristic curves were computed on each significant symptom, and Youden indexes were calculated to determine the critical level of symptomatology at which the performance significantly changed.

**Results:** Results show that 97.7% of patients who presented with LID during the experiment also presented with at least one cardinal motor symptom. On average, patients took more time and did more errors during ADL. Multivariate analyses revealed that for the great majority of ADL, LID were not associated with worsening of performance; however, postural instability, tremor, rigidity, and cognitive decline significantly decreased the odds of success.

**Conclusions:** Residual symptoms of PD, such as tremor, rigidity, and postural instability still present at peak-dose were more problematic than LID in the performance of ADL for patients experiencing slight-to-moderate LID. We also found that cognitive decline was associated with decreased performance in certain tasks. Therefore, a strategy using lower doses of medication to manage LID may be counterproductive since it would not address most of these symptoms already present in patients.

## Introduction

Chronic levodopa therapy combined with the further progressing loss of dopaminergic neurons in the substantia nigra pars compacta (1) will lead to pre- and post-synaptic plasticity (2, 3), resulting in motor complications called levodopa-induced dyskinesia (LID). Currently, there is no clear agreement in the literature on the impact of the severity of LID on activities of daily living (ADL) and on the quality of life (QoL) of patients. While some studies suggested that LID impact mobility and has stigma associated with it (4–7), others highlighted the impact of other motor dysfunctions like tremors, gait, and mobility impairments (8–14), and cognition (6, 15) on ADL and QoL. The great majority of those studies used questionnaires to assess the symptomatology as well as ADL and QoL, which can be impacted by recall issues, anosognosia (16–18), depression or anxiety (15, 19, 20). To counteract these issues, some studies used a more quantitative approach where motor performance of patients having LID was compared with those of patients without LID and healthy controls in manual tracking tasks requiring high level of precision (21, 22). They showed that patients having LID had more errors and a poorer motor control than other groups. However, the authors did not measure LID during the motor activity to assess their relationship with observed errors. In a similar study, authors measured LID during the manual tracking task, but they did not find any relationship with the observed errors, suggesting that other motor symptoms, not measured in this study, could have played a role in the errors shown in patients (23). Not long ago, we demonstrated that patients having LID could also experiment other motor symptoms such as tremor, bradykinesia, rigidity, or postural instability concomitantly with peak-dose LID (24).

In order to provide a more definite answer on whether LID has an impact on motor performance associated with ADL, it becomes imperative that we assess the level of LID while patients are performing these daily tasks. Also, we need to consider the presence of other symptoms since they may be more problematic for patients than LID. Recently, it was proposed that the impact of LID and cardinal motor symptoms could be viewed as a function of a signal-to-noise ratio (SNR) (25), where the signal would be the intended voluntary movement and the noise would be the symptomatology interfering with the voluntary movement. This SNR approach is illustrated by the theoretical formula below.

$$\begin{array}{l} SNR=\frac{Voluntary\;movement}{Tremor+Bradykinesia+Postural\;instability+Rigidity+Cognition+DID} \end{array}$$

Here, the impact of symptomatology is directly related to the type of voluntary movement performed by patients, as well as the predominance of specific symptoms. For example, a task with low amplitude of displacement and velocity, which are required during fine dexterous movements, could be more influenced by noises such as tremor or LID. In contrast, a task with high amplitude of displacement or velocity could be more affected by bradykinesia or postural instability. Based on these assumptions, it would then be possible to determine which symptom(s) impacts negatively on the performance of the task itself, based on the level of symptomatology present while performing the task.

Therefore, the aims of this study were to (1) measure objectively symptoms that are present concomitantly with LID during ADL using a motion capture system; (2) determine which symptoms impact the performance of ADL executed at peak-dose; (3) determine the critical amplitude of symptoms at which the performance becomes deteriorated. Even if LID can take place at several moment during the pharmacodynamic of levodopa, peak-dose dyskinesia is the most common type (26–28). Therefore, we only focused on choreic-type LID occurring at peak-dose.

## Materials and Methods

### Participants

One hundred and twenty-one patients diagnosed with idiopathic Parkinson's disease according to the UK Parkinson's Disease Society Brain Bank clinical diagnosis criteria (29) were recruited in this cross-sectional study through the Quebec Parkinson Network and clinicians specialized in movement disorders. Patients selected in this study were known to have experienced clinically-detectable choreic-type LID in the past months prior to the experiment. Patients requiring assistance to walk or having orthopedic conditions that could hinder the performance of required tasks were not considered for the study. Data were collected in three Canadian research institutes: the Centre de Recherche de l'Institut Universitaire de Gériatrie de Montréal, the Institut de Réadaptation en Déficience Physique de Québec, and the Movement Disorders Program of the University of Calgary, from June 2016 until June 2017. 69 healthy control participants were also recruited through the Centre de Recherche de l'Institut universitaire de gériatrie de Montréal to provide a “normal” baseline of behavior during the performance of each ADL. Healthy control participants were aged between 45 and 85 years old, and were able to walk without assistance. Exclusion criteria included any orthopedic condition that could hinder the execution of ADL or be under the influence of any medication that could cause issues with concentration and involuntary movements. The study protocol was approved by the Comité d'éthique de la recherche vieillissement-neuroimagerie of the Centre de Recherche de l'Institut universitaire de gériatrie de Montréal for data that were collected in Montréal (CER IUGM 13-14-022), by the Comité d'éthique de la recherche sectoriel en réadaptation et integration sociale for data that were collected in Quebec (MP-2016-510), and by the Conjoint Health Research Ethics Board of University of Calgary for data that were collected in Calgary (REB 16-2551). Each participant read and signed an informed consent form. Part of the data from patients used in the present study originated from another study on the concomitant presence of LID and cardinal motor symptomatology (24).

### Experimental Procedures

**Figure 1:**
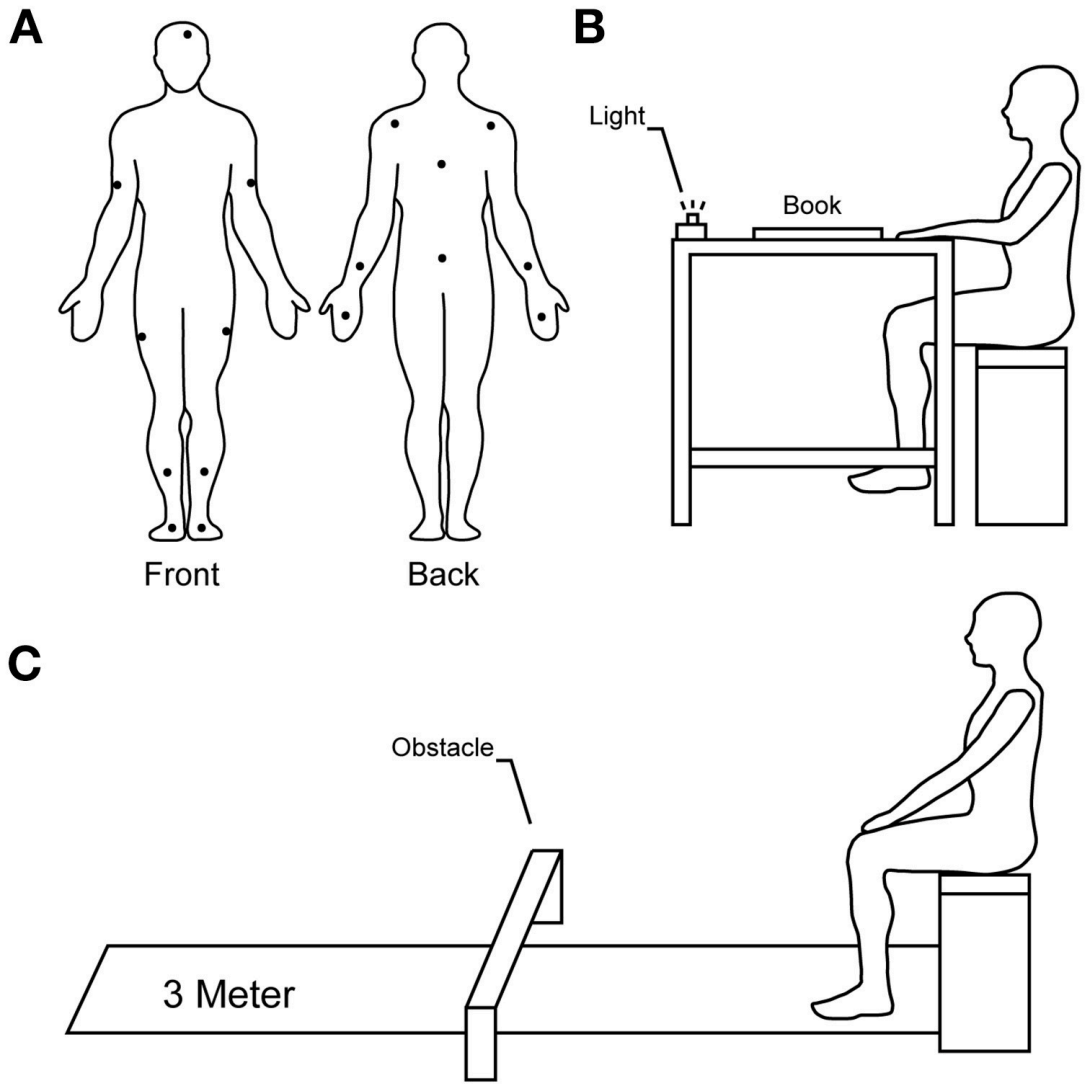
Sensors placement and spatial schematics of the experimentation. **(A)** Diagram illustrating the position of inertial sensors on the body of participants. **(B)** Starting position of sitting ADL. **(C)** Starting position of TUG tasks.

Patients' visit to the laboratory was usually planned in the afternoon, coinciding with their scheduled medication dose deemed to generate the highest LID amplitude. They were instructed to take their medication at their usual time of day when arriving at the laboratory, coinciding with the administrative requirements for the study participation, and the administration of the Mini-Mental State Examination (MMSE). A quantitative approach was favored to assess the levels of LID, tremor, bradykinesia, and freezing of gait (FoG). To do so, patients were equipped with a motion capture suit (Animazoo IGS-180, Synertial UK Ltd) with 17 inertial measurement units positioned on each body limb. Each Unit contained a 3-axis accelerometer, a 3-axis gyroscope, and a 3-axis magnetometer (Figure 1A). We previously tested the accuracy of these sensors (30, 31), developed automated quality control (32) and segmentation algorithms to assess the mobility of elderly (33–35) and patients with Parkinson's disease (36, 37).

Data collection began as soon as the investigators visually noticed choreic-type LID. The testing period proceeded in two blocks of tasks, during which investigators regularly asked patients for feedback about whether they felt that the medication reached their most efficient stage, based on their experience. The first block was used for the assessment of some motor symptoms of Parkinson's disease such as bradykinesia, postural instability, and rigidity. Core bradykinesia, defined as bradykinesia that is not the results of a strategy to avoid errors (38), was assessed using a rapid alternating movement (RAM) task described elsewhere (39–41). In brief, patients were instructed to perform pronation-supination movements of the forearm with maximal rotational amplitude and maximal velocity for 10 s. The outcome measure of the RAM task was the averaged maximal angular velocity of each cycle of pronation-supination movements (38, 42–44). Postural instability and rigidity were both assessed using the MDS-UPDRS item 3.12 and item 3.3, respectively, since efficient objective assessment of these symptoms is not yet available. Block 1 was repeated three times.

The second block, performed in between blocks 1, was composed of a series of ADL selected from a comprehensive list of ADL scales and questionnaires. Hyperkinetic movements such as LID, tremor, as well as FoG were assessed during these ADL.

Tasks were selected by going through several ADL questionnaires, including some specific to Parkinson's disease. To be selected in our protocol, a task had to meet three criteria: (a) be a motor task that is performed during daily life activities to avoid practice effect, (b) be able to set clear criteria on whether the task was a success or not, and (c) be able to quantify the performance using our inertial motion capture system. After eliminating redundancies and applying our criteria, our exercise yielded 31 possible ADL. Pilot testing allowed us to identify 11 ADL having different amplitude of displacement and velocity profiles. ADL included in this study were *reading, counting money, cutting*-*eating* a piece of apple, *eating soup, taking medication* (sugar pills), and *drinking water*. Patients performed these tasks while sitting down on a bench with both hands positioned on a table (Figure 1B). Once cued by a light positioned in front of them, patients were instructed to perform the ADL at their preferred pace to reproduce true living conditions. Once each ADL was completed, patients were asked to return their hands to their original position.

For *reading*, patients had to take a book positioned on the table, open it at the marked page and read at loud one of the two proposed text composed of 10 lines each. When patients finished reciting the text, they were instructed to close the book and to return it on the table. Patients were instructed to hold the book in their hands without resting their arms on the table while reading. For *counting money*, patients had to take a cup containing an unknown amount of coins, empty the content into their hands, count the amount of money, and put back the coins in the cup. Patients were instructed to keep coins in their hands while counting. The amounts of money (4.60$ CAD and 3.35$ CAD) were different for the two trials to avoid a memory effect that could affect the results, and the order of presentation was randomized. The number and type of coins used for each amount were similar for all patients (1 two-dollar, 1 dollar, 5 quarters, 3 dimes, 1 nickel, and 1 two-dollar, 4 quarters, 3 dimes, 1 nickel, respectively, for the two amounts cited above). For *cutting-eating*, patients had to take the fork and the knife positioned on the table, cut a piece of apple into four pieces, eat two pieces using the fork, and return the cutlery on the table. The prepared piece of apple was the same size and same shape for each patient. For *eating soup*, patients had to take the spoon positioned on the table, take four spoons of water, and return the spoon on the table. For *taking medication*, patients had to take the pill organizer containing a candy, open it, take the candy, put it in their mouth, close the pill organizer, and return it on the table. For *drinking water*, patients had to take a glass full of water with their dominant hand, drink two separate sips, and return the glass on the table.

ADL also included three different Timed-Up and Go (TUG) tasks; a 3-meter *TUG*, a 3-meter TUG while carrying a glass full of water (*TUG-GW*), and a 3-meter TUG with an obstacle to step over (*TUG-O*). The obstacle was placed at 1.5-meter from the starting position. The dimension of the obstacle was 465 mm long, 150 mm wide and 250 mm high. Patients began in a sitting position on the bench (Figure 1C). Once cued by the light, patients rose from the bench they were sitting, without help from their hands, performed the TUG and returned to their original position. Finally, patients performed a *sitting-standing* task without help from their hands to stand and sit. Lastly, patients were asked to *pick*-*up an object* off the floor. In this task, patients stood in front of the light with an eraser placed at 50 centimeters from their feet. Once cued by the light, they were asked to pick-up the object with their dominant hand and raise it at their eyes level.

The time required to perform each task was calculated between the light stimuli and the moment where patients reached their final position. The number of errors was calculated for each task. Error criteria included: dropping an object, dropping water, needing assistance, or multiple trials to rise from the bench, needing assistance to sit on the bench, needing assistance to walk or to step over the obstacle, touching the obstacle, or omitting to complete some procedures.

The tremor level, referring in this study to all kind of tremors (rest, postural, or kinetic), was assessed during the performance of ADL using similar methodology employed in the past by us and others (45–49). Signals from gyroscopes (x, y, z) positioned on the hands were band-pass filtered between 3.5 and 7.5 Hz to remove the voluntary movements and the noise, detrended and segmented in 5 s windows. For each window, the power spectral density was calculated for the x, y, and z directions. These components were summed to generate the total power spectral density. The dominant frequency was identified, and the power dispersion, representing the width of a frequency band containing 68% of total power centered around that dominant frequency was calculated. A narrow dispersion (i.e., below 2 Hz) is indicative of a dominant oscillation normally associated with abnormal tremor. If present, the amplitude of tremor was calculated by computing the power estimation within the frequency band identified by the power dispersion calculation. If the dispersion around the dominant frequency was more than 2 Hz, the spectrum was visually verified to ensure the absence of a sharp peak of tremor. The tremor level of each window was then summed to generate a tremor level for the entire task.

The levels of choreic-type LID were also measured while performing these ADL using a methodology inspired by the literature (50–54). For sitting ADL, LID was quantified using all sensors, except those positioned on the arms that were directly involved in voluntary movements. For standing ADL, LID was quantified using all sensors, except those positioned on the legs, which were involved in ambulation. Here we chose to exclude these sensors since voluntary movements and LID are in the same frequency bandwidth. This technique represents a compromise between including all LID and having a lot of component of voluntary movements, and excluding some LID in our measure in excluding the majority of voluntary movements that could lead to erroneous interpretation of results. Because LID are defined as involuntary, irregular, purposeless, non-rhytmic, abrupt, rapid, unsustained movements that seem to flow from one part of the body to another, affecting the neck, the trunk, and the limbs (55), we think that eliminating some LID in excluding voluntary movement will still give a good approximation about the level of LID in the body. Signals from gyroscopes (x, y, z) were band-pass filtered between 0.5 and 4 Hz to remove the noise, and then detrended. The magnitudes of the coordinate vectors (x, y, z) were computed at each frame resulting in new time series. These new time series were then segmented in 1 s windows and power spectral densities were computed on each window. The power dispersion around the dominant frequency was verified to ensure that dyskinesia was not being confounded with tremor, before calculating energy spectrums. The mean energy was calculated for each segment of each window (3 sensors for each leg, 3 sensors for each arm, 4 sensors for the trunk, 1 sensor for the head). Then, for each segment, we calculated the 75th percentile plus 1.5 inter quartile range threshold, and we considered each point above that threshold as a possible outlier resulting in a superfluous, isolated voluntary movement. Outliers were visually verified by video analysis and were reset to the highest value in the acceptable range only if a superfluous voluntary movement was seen. The mean of each 1 s window of each segment was then calculated and the sum of segments was calculated, given an average energy value per second, per task.

FoG is known to impact ADL and QoL and to be often resistant to levodopa treatment (8). FoG was assessed during TUG tasks using an algorithm proposed by Moore et al. (56). In brief, FoG results from an absence of forward locomotion and high frequency trembling of the lower limbs occurring in the 3–8 Hz bandwidth, whereas walking is characterized by a power spectrum under 3 Hz. Therefore, a ratio between the power of the freezing bandwidth and the walking bandwidth is used on linear acceleration of multiple sensors (feet, shanks, thighs, and hip) to determine when participants are in a frozen state. To be in a frozen state, this ratio should be above the threshold determined by Moore et al. (56) for at least four of the seven sensors used in this algorithm. The percent time frozen during walking was calculated for each TUG task.

The order of symptomatology assessment within Block 1 remained the same, but the order of ADL was randomized within Block 2. Block order presentation remained the same for all participants, i.e., Block 1—Block 2—Block 1—Block 2—Block 1. This design enabled us to select ADL tasks and symptomatology assessments when patients were best ON, meaning when LID was at its highest amplitude. For each recorded ADL, we used the levels of bradykinesia, rigidity, postural instability, and FoG that were the closest in time (i.e., ± 10 min) as outcome measures. Block 1 lasted about 10 min while block 2 lasted about 15 min. The entire recording session lasted ~1 h.

### Statistical Analyses

First, we identified patients who experienced LID during the experiment by comparing the maximum amplitude of each patient's LID during movement with a threshold set using movements of healthy controls (mean + 1SD), giving a high level a sensitivity (91%) and specificity (87%). This first selection of patients having LID was compared with annotations written during the experiment and with video recording of the testing sessions, and groups were manually readjusted to avoid false positive and false negative.

For each ADL, trials where patients had the highest levels of LID were identified. Performance of ADL (time required to perform the ADL and number of errors) was compared between healthy controls and patients using non-parametric Kruskal-Wallis tests.

To identify the origin of the observed differences in the performances between healthy controls and patients, a success criterion was set for each task based on the mean plus two standard deviations of controls' performance for time and errors. Then, a binomial logistic regression was performed to assess the impact of each symptom on the success of each ADL. A purposeful selection method (57) of factors (i.e., LID, bradykinesia, tremor, rigidity, postural instability, FoG, age, and cognitive status measured by MMSE) was used for the different models. Briefly, the purposeful selection method begins with a univariate analysis of each outcome measure. Any outcome measure having a significant univariate test is selected in the multivariate analysis. Any non-significant variable having a confounding effect of 15% or more on the parameter estimate is added back in the multivariate analysis. At the end, the model contains only significant variables and confounders.

Finally, receiver operating characteristic (ROC) curves and ensuing Youden indexes were used on significant outcome measures to highlight the critical point at which failure in performing tasks within success parameters occurred. Analyses were done using R (R Foundation for Statistical Computing, Vienna, Austria). All statistical significance thresholds were set at α < 0.05.

## Results

**Figure 2:**
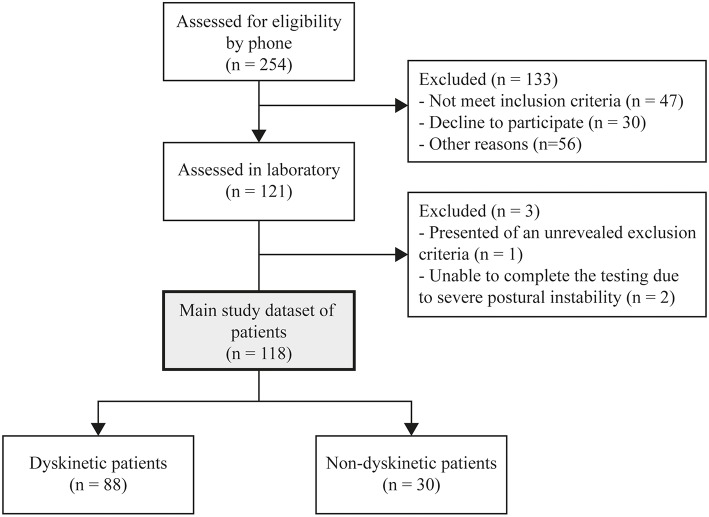
Flowchart of the inclusion of participants.

From the 121 patients recruited for the study, three were excluded from the analyses; one presented with severe osteoarthritis, two others did not complete the experiment due to severe postural instability. Eighty-four of the remaining patients reached our threshold of LID. After examining questionable cases where movement patterns did not seem to meet the characteristics of peak-dose choreic-type LID, four patients were identified as false-positive (voluntary movements detected as LID) and were excluded from further analyses, while eight false-negatives were added back to the sample of patients having LID following video analyses. This yield to a total of eighty-eight patients with peak-dose choreic-type LID during the experiment (74.6%). Patient selection flowchart and clinical profile are shown in Figure 2 and Table 1, respectively.

**Table 1:** Characteristics of controls and patients having LID.

**Characteristics**	**Mean ± SD**	**Range**
***Controls (n = 69; 30 Females)***		
Age (year)	68.1 ± 7.7	45–82
MMSE (/30)	28.6 ± 1.5	23–30
GDS-15 (/15)	1.7 ± 2.0	0–11
***Patients (n = 88; 41 Females)***		
Age (year)	67.5 ± 8.7	37–83
MMSE (/30)	27.3 ± 2.5	19–30
GDS-15 (/15)	3.7 ± 2.8	0–13
Years since diagnosis	10.4 ± 5.5	1–30
LEDD (mg)^a^	1048.9 ± 520.0	200–2,790
**MDS-UPDRS PART III ON**^b^		
Speech	1.2 ± 1.0	0–4
Facial expression (3.2)	1.6 ± 1.0	0–4
Arms rigidity (3.3)^c^	0.7 ± 0.7	0–2.5
Legs rigidity (3.3)^c^	1.1 ± 0.8	0–4
Arising from chair (3.9)	0.4 ± 0.7	0–3
Gait (3.10)	1.1 ± 0.9	0–3
Freezing of gait (3.11)	0.3 ± 0.7	0–4
Postural instability (3.12)	1.2 ± 1.0	0–4
Posture (3.13)	0.8 ± 0.9	0–3
Bradykinesia (3.14)	1.0 ± 1.0	0–4
Postural tremor (3.15)^c^	0.4 ± 0.8	0–4
Rest tremor (3.17)^c^	0.2 ± 0.5	0–3
**MDS-UPDRS PART IV**^b^		
Time spent with LID (4.1)	1.6 ± 0.9	0–4
Functional impact of LID (4.2)	1.7 ± 1.0	0–4
Hoehn and Yahr score ON^b^	2.3 ± 0.7	1–4

a *Missing data for 9 participants*.

b *Higher score indicates worse functioning*.

c *Score represent the mean of the left and right segments*.

*GDS-15, 15-item Geriatric Depression Scale; MDS-UPDRS, Movement Disorder Society-Unified Parkinson's Disease Rating Scale; SD, Standard deviation, LEDD, Levodopa Equivalent Daily Dose*.

In this group of patients, 97.7% presented at least one other motor symptom concomitantly with LID; 25.0% presented with bradykinesia, 12.5% with tremor, 63.6% with mild or intermittent rigidity, 71.6% with postural instability, and 6.8% with FoG.

**Figure 3:**
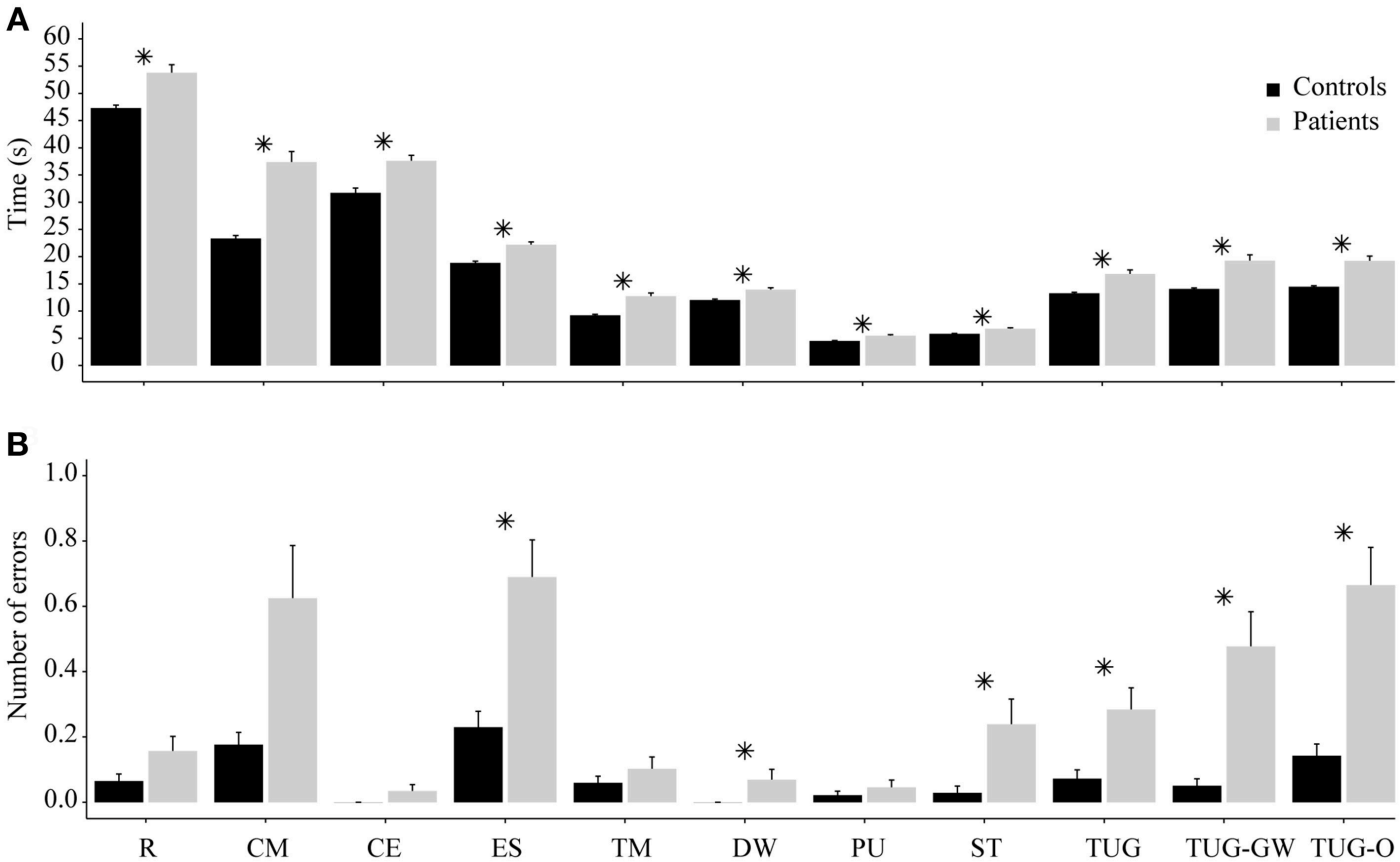
Performance comparison between controls and patients. **(A)** Comparison of time required in each ADL between patients and controls. **(B)** Corresponding errors made in each ADL between patients and controls. R, *Reading*; CM, *Counting money*; CE, *Cutting-eating*; ES, *Eating soup*; TM, *Taking medication*; DW, *Drinking water*; PU, *Pick-up an object*; ST, *Sitting-standing*; ^*^means statistically significant; error bars correspond to standard error.

Kruskal-Wallis tests revealed that patients having LID were significantly slower than controls to perform sitting and standing ADL (*reading*: H(1) = 9.10, *p* < 0.01), *counting money*: [*H*_(1)_ = 42.00, *p* < 0.01], *cutting*-*eating*: [*H*_(1)_ = 20.99, *p* < 0.01], *eating soup*: [*H*_(1)_ = 21.12, *p* < 0.01], *taking medication*: [*H*_(1)_ = 26.10, *p* < 0.01], *drinking water* [*H*_(1)_ = 13.77, *p* < 0.01], *pick-up an object*: [*H*_(1)_ = 15.41, *p* < 0.01], *sitting-standing*: [*H*_(1)_ = 12.50, *p* < 0.01], *TUG*: [*H*_(1)_ = 23.51, *p* < 0.01], *TUG-GW*: [*H*_(1)_ = 36.02, *p* < 0.01], *TUG-O*: [*H*_(1)_ = 19.95, *p* < 0.01] (Figure 3A). Also, patients had more errors than controls only in *eating soup* [*H*_(1)_ = 5.30, *p* = 0.02], *drinking water* [*H*_(1)_ = 4.07, *p* = 0.04], *sitting*-*standing* [*H*_(1)_ = 7.40, *p* < 0.01], *TUG* [*H*_(1)_ = 5.58, *p* = 0.02], *TUG-GW* [*H*_(1)_ = 12.92, *p* < 0.01], *TUG-O* [*H*_(1)_ = 9.16, *p* < 0.01] (Figure 3B). Age and gender proportion was equal between groups.

Results from the multivariate analysis are presented in Table 2. For each ADL, we report the significant variables in the univariate testing and/or those inducing a change in any parameter estimate >15% as compared to the model (57). An odds ratio greater than 1 means that greater the score/value of symptoms, greater is the performance. In the opposite way, an odds ratio lower than 1 means that greater the score/value of symptoms, lower is the performance. They revealed that rigidity had a negative impact on *eating soup*, tremor had a negative impact on *counting money* and *TUG-GW*. Furthermore, postural instability had a negative impact on *picking-up an object, TUG, TUG-GW*, and *TUG-O*, and LID only had a negative impact on *eating soup*. Multivariate analyses also showed that the odds of success in *reading, counting money, TUG*, and *TUG-O* were lower for patients with a lower score of MMSE. Conversely, the odds of success in *taking medication, TUG*, and *TUG-GW* were higher for patients with a higher level of LID.

**Table 2:** Results of binary logistic regressions.

**Task selected variables**	**Odds ratio (95% CI)**	***p*-values**	**Effect on the odds of failure**
***Reading***			
Postural instability	0.64 (0.38; 1.05)	0.081	
MMSE	1.41 (1.14; 1.82)	**0.004**	Increase
***Counting money***			
Postural instability	1.61 (0.94; 2.98)	0.101	
Bradykinesia	1.00 (0.1000; 1.003)	0.057	
Tremor	0.76 (0.60; 0.90)	**0.006**	Increase
MMSE	1.38 (1.09; 1.82)	**0.013**	Increase
***Cutting-eating***			
Bradykinesia	1.00 (0.999; 1.001)	0.394	
Age	0.97 (0.92; 1.03)	0.387	
MMSE	1.06 (0.87; 1.29)	0.577	
***Eating soup***			
LID	0.80 (0.66; 0.92)	**0.008**	Increase
Bradykinesia	1.00 (0.999; 1.003)	0.154	
Rigidity	0.48 (0.23; 0.95)	**0.041**	Increase
Tremor	0.33 (0.07; 0.90)	0.068	
***Taking medication***			
LID	1.57 (1.18; 2.41)	**0.014**	Decrease
Bradykinesia	1.00 (0.999; 1.003)	0.154	
Tremor	0.47 (0.19; 1.00)	0.067	
Rigidity	0.80 (0.41; 1.55)	0.501	
FoG	0.05 (6e-11; 630)	0.680	
Age	0.98 (0.91; 1.04)	0.472	
MMSE	1.21 (0.94; 1.58)	0.158	
***Drinking water***			
Bradykinesia	1.00 (1.000; 1.004)	0.057	
Age	0.96 (0.88; 1.04)	0.372	
MMSE	1.11 (0.88; 1.40)	0.354	
***Picking-up an object***			
LID	1.02 (0.98; 1.08)	0.418	
Bradykinesia	1.00 (0.999; 1.004)	0.064	
Postural instability	0.44 (0.23; 0.78)	**0.007**	Increase
FoG	0.01 (2e-9; 4.26)	0.326	
Age	0.98 (0.90; 1.06)	0.648	
MMSE	1.09 (0.86; 1.40)	0.479	
***Sitting-standing***			
Postural instability	0.67 (0.43; 1.02)	0.064	
***TUG***			
LID	1.04 (1.01; 1.09)	**0.034**	Decrease
Bradykinesia	0.10 (0.998; 1.001)	0.321	
Tremor	0.93 (0.83; 1.01)	0.126	
Postural instability	0.35 (0.17; 0.62)	**0.001**	Increase
Age	0.95 (0.88; 1.03)	0.246	
MMSE	1.58 (1.17; 2.28)	**0.007**	Increase
***TUG-GW***			
LID	1.15 (1.05; 1.27)	**0.005**	Decrease
Bradykinesia	1.00 (0.998; 1.001)	0.873	
Tremor	0.64 (0.40; 0.97)	**0.048**	Increase
Rigidity	0.71 (0.34; 1.43)	0.348	
Postural instability	0.44 (0.22; 0.80)	**0.012**	Increase
Age	0.97 (0.90; 1.05)	0.489	
MMSE	1.33 (1.01; 1.83)	0.060	
***TUG-O***			
LID	1.03 (1.00; 1.08)	0.066	
Bradykinesia	1.00 (0.998; 1.001)	0.662	
Tremor	0.93 (0.84; 1.00)	0.092	
Postural instability	0.48 (0.25; 0.82)	**0.013**	Increase
Age	0.98 (0.91; 1.05)	0.498	
MMSE	1.34 (1.04; 1.81)	**0.037**	Increase

*Bold values indicate statistically significant (α = 0.05)*.

**Figure 4:**
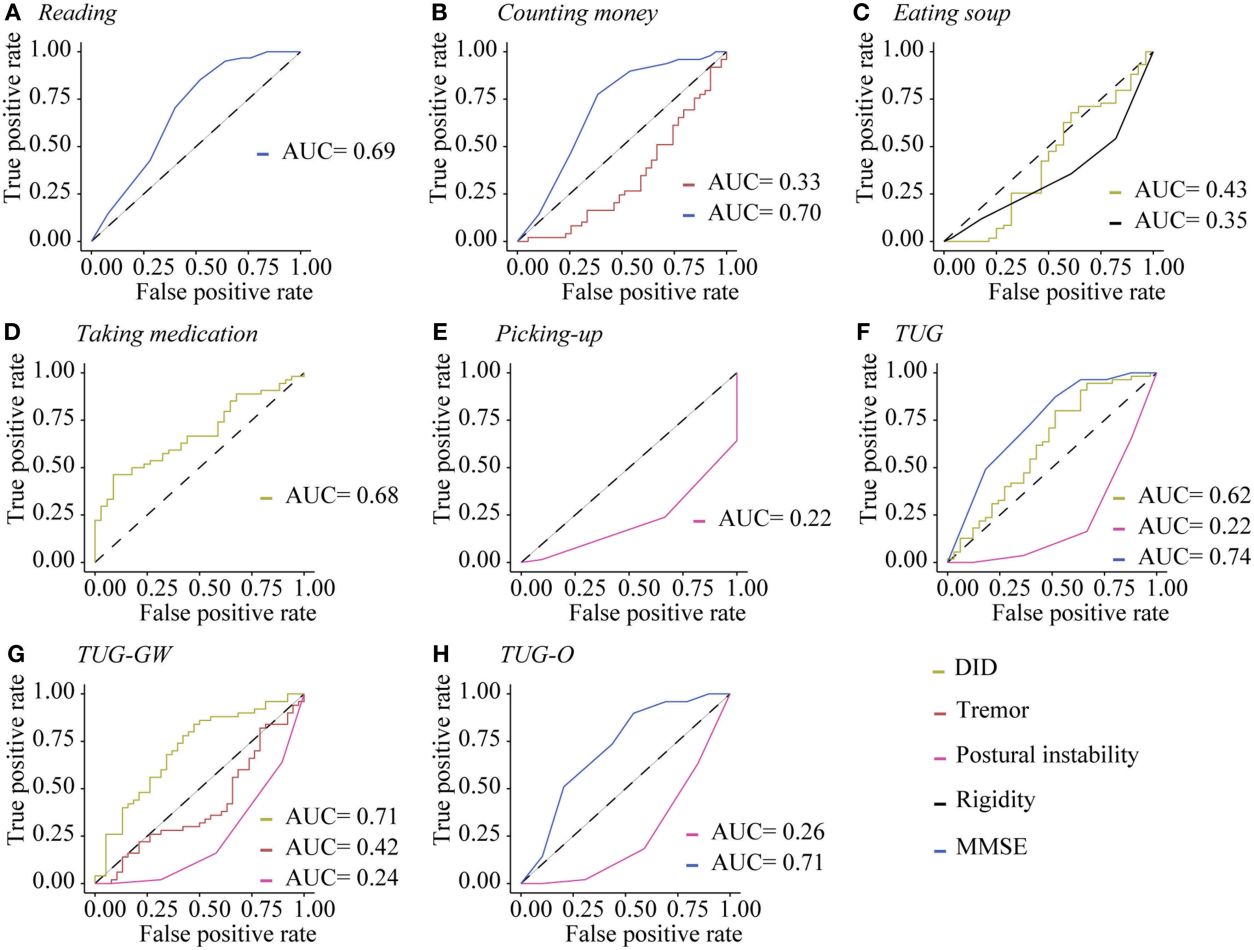
ROC curves of significant factors impacting the performance of ADL. ROC curves of significant variables for **(A)** *reading*, **(B)** *counting money*, **(C)** *eating soup*, **(D)** *taking medication*, **(E)** *picking-up an object*, **(F)** *TUG*, **(G)** *TUG-GW*, **(H)** *TUG-O*. AUC, Area Under the Curve.

ROC curves analyses (Figure 4) and Youden indexes (Table 3) show that patients with an MMSE score below than 28 had higher odds of failure in *reading, counting money, TUG*, and *TUG-O*. Patients with high level of postural instability had higher odds of failure in standing ADL. Patients with a rigidity score higher than 2.5 had higher odds of failure in *eating soup*. A tremor level higher than 0.5 increased the odds of failure in *TUG-GW*, and a tremor level higher than 3 increased the odds of failure in *counting money*. Plus, patients with higher level of LID (>7) had higher odds of failure in *eating soup*, but they had higher odds of success in *taking medication, TUG*, and *TUG-GW*.

**Table 3:** Critical values of symptoms at which the performance becomes affected.

**Significant variables**	**Area under the curve**	**Youden index**	**Clinical meaning of youden index**
***Reading***			
MMSE	0.69	28	MMSE < 28
***Counting money***			
Tremor	0.33	2.33	Tremor > 1.5^a^
MMSE	0.70	28	MMSE < 28
***Eating soup***			
LID	0.42	4.33	LID > 7^a^
Rigidity	0.35	2.5	Rigidity > 2.5
***Taking medication***			
LID	0.68	3.25	LID < 4^a^
***Picking-up an object***			
Postural instability	0.22	4	Postural instability > 2
***TUG***			
LID	0.62	37.94	LID < 3^a^
Postural instability	0.22	4	Postural instability > 2
MMSE	0.74	28	MMSE < 28
***TUG-GW***			
LID	0.71	18.27	LID < 2^a^
Tremor	0.42	1.68	Tremor > 0.5^a^
Postural instability	0.24	4	Postural instability > 2
***TUG-O***			
Postural instability	0.26	4	Postural instability > 2
MMSE	0.71	28	MMSE < 28

a *Correspond to the sum of UDysRS or UPDRS scores of each limb assessed by a trained examiner*.

## Discussion

Results from the present study demonstrate that some patients presenting with peak-dose choreic-type LID also exhibited cardinal motor symptoms associated with Parkinson's disease. In general, patients were significantly slower than controls to complete the required tasks, and demonstrated significantly more errors in the *eating soup, drinking water*, and *TUG* tasks. Interestingly, these differences were not due to LID. In fact, patients with a higher level of LID seemed to have higher odds of success in some ADL, and LID was only deleterious in one of the ADL (*eating soup*). On the other hand, patients with higher residual cardinal motor symptoms still present at peak-dose such as tremor, postural instability, or rigidity, or with a lower cognition level, had significantly higher odds of failure in some ADL.

To our knowledge, it is the first time that LID, other symptoms and motor performance are assessed simultaneously in such a large cohort, providing clear evidence of the lack of impact of slight-to-moderate LID on ADL. These results refute the negative impact of LID in ADL proposed by others through the evaluation of questionnaires data (4–7), but highlight the negative effects of other symptoms, such as postural instability, tremor, rigidity, and decline in cognition (8–14). These results also show that the impact of symptoms depend on the activities performed by patients. Clearly, LID had a negative impact only on the task requiring the highest precision, i.e., *eating soup*. For other ADL, the SNR was probably high enough to perform the activities. Despite our effort to recruit patients with a wide range of LID amplitude, it is important to note that our participants had mostly slight-to-moderate LID (*n* = 80 patients with a score ranging from 1 to 3 in the MDS-UPDRS item 4.2), meaning that the great majority of them were well-managed. Clearly, patients recruited for this study presented with “functional” LID, instead of “dysfunctional” LID where the SNR would be too low to perform the task efficiently. There may be an amplitude threshold at which LID would become “dysfunctional”; finding this threshold may serve as a marker to contemplate more aggressive treatment such as surgery to manage LID. One of the goals of this study was to find the threshold at which any pertinent symptom would induce failure in performing a specific ADL according to our criteria. For LID, only the *eating soup* task generated such threshold. Nevertheless, we were able to find clinical thresholds for MMSE, rigidity, tremor and postural instability scores, this for several ADL. These results suggest that the mere presence of rigidity, tremor, postural instability or cognitive signs, for example, is not necessarily problematic for patients, unless the magnitude of these symptoms reach a certain level. Further research will be needed to utilize those thresholds to predict the motor repertoire available of patients based on their symptomatology.

In this study, the failures in ADL were mainly caused by postural instability for walking tasks, tremor, and rigidity for tasks requiring precision like transporting a glass of water (*TUG-GW*) or *counting money*, and decline in cognition for tasks requiring higher cognitive implication such as *reading, counting money*, or walking tasks which require attention (58, 59). It is then reasonable to suggest that a strategy using lower doses of medication to manage LID may be counterproductive since it may aggravate symptoms already present in patients. In fact, according to our SNR model, such strategy would have the potential to increase the noise level, hence reducing the SNR. This study also supports our previous (25) suggestion that success in managing LID should be based on the motor repertoire available of patients, not on the amplitude of LID, at least in the slight-to-moderate cases of LID.

It is important to note that while slight-to-moderate LID seemed not to have a deleterious effect on the ADL selected in this study, it remains a stigma that can affect the well-being of patients and consequently interact in their ADL (4–7). This fact must be taken into consideration when managing LID in these patients. In this study, the goal was to assess the true ability of patients to perform ADL while experiencing LID. To do so, we used success criteria objectively based on the performance of healthy control participants. A weakness of the present study is that we did not ask the patients' insights about their comfort while performing the ADL, and for their self-rated estimation of disability during ADL. This could have provided additional information about their well-being while performing ADL, reinforcing the results of this study.

To our knowledge, the current experiment represents the largest full-body kinematic study of patients with Parkinson's disease having LID; allowing us to capture the true amplitude of LID throughout ADL, as well as most cardinal symptoms using inertial sensors data. Even if some symptoms were sequentially measured before and after ADL, additional analysis revealed no significant differences between symptomatology levels between different blocks. Therefore, the sequential measure of some symptoms had no impact on the interpretation of results.

Furthermore, this protocol used daily life tasks, avoiding practice effects or task novelty, but also highlighted the symptomatology patients live with every day. This methodology allowed us to directly relate ADL motor performance of patients with the symptomatology present during the testing. Consequently, we now have a better idea about the true ability of patients in performing motor tasks while experiencing LID, rather than rely on questionnaire assessment biased by depression, anxiety (15, 19, 20) or anosognosia (16, 17). Indeed, we think that the mood and the anosognosia did not influence the motor performance of patients in our study because they accepted to perform the required tasks, even with the presence of dyskinesia. This constitutes a novelty that can bring the debate about the impact of LID on the ADL to the next level, and we think that this provides the principle force of this study. We did not use a non-dyskinetic control group for the following reasons: first we specifically wanted to assess the influence of the symptomatology on ADL performance in patients with LID. Second, determining ‘peak-dose’ of patients without dyskinesia is difficult because of the absence of a telltale signs like dyskinesia. If we would have tested non-dyskinetic patients, it is quite reasonable to assume that some of these patients could have been tested at other point in time in their pharmacodynamic curve, which would have rendered the interpretation of the results extremely difficult.

We use the term LID in the present study, but it is noteworthy that few patients were treated solely with Levodopa, and that dopamine agonists may have also participated in the generation of these involuntary movements. Since we excluded LID occurring in the performing limb, it is quite possible that we have somewhat underestimated the amplitude of involuntary movements. However, because of the nature of choreic-type LID that seem to flow from one part of the body to another (55), this limitation does not weaken our results. A limitation of this study is that we were not able to measure plasma levels of levodopa during the experiment to confirm the moment at which peak-dose was reached. Nevertheless, this moment usually coincides with the period during which maximal amplitude of choreic-type LID is present, from which our data originated. Another limitation of the study was due to the somewhat restricted variability of LID amplitude present in our sample. This is why our results only apply to patients with slight-to-moderate LID. It is reasonable to think that for each ADL performed in this study, there may be an amplitude threshold at which LID would become problematic. Unfortunately, patients with severe amplitude of LID were rare in our sample. Therefore, determining that threshold was not possible. This issue should be addressed in the future.

In conclusion, the present study demonstrates in a quantitative manner that residual symptoms of Parkinson's disease still present at peak-dose are more problematic than LID in the performance of specific ADL for patients experiencing slight-to-moderate peak-dose choreic-type LID.

## Data Availability

The datasets used and/or analyzed during the current study are available from the corresponding author on collaborative work.

## Author Contributions

EG was responsible for the development of the protocol, data collection, data analyses, and drafted the manuscript. HN and SB were involved in the data collection and provided significant feedbacks on the analysis of the study. PB, EB, and CV were involved in the conception and design of the study and critical revision of manuscript for intellectual content. JS and OM were involved in the data collection and critical revision of manuscript for intellectual content. CD, the lead scientist helped in all facets of the project. All authors read and approved the final manuscript.

### Conflict of Interest Statement

EB has equity stake in Motac holding Ltd. and receives consultancy payments from Motac Neuroscience Ltd., companies which pre-clinical activity has no relationship with the present study. The remaining authors declare that the research was conducted in the absence of any commercial or financial relationships that could be construed as a potential conflict of interest.

## Abbreviations

ADL: Activities of daily living
LID: Levodopa-induced dyskinesia
FoG: Freezing of gait
GDS-15: 15-item geriatric depression scale
LEDD: Levodopa equivalent daily dose
MMSE: Mini-mental state examination
QoL: Quality of life
RAM: Rapid alternating movement
ROC: Receiver operating characteristic
SNR: Signal-to-noise ratio
TUG: Timed-up and go
TUG-GW: Timed-up and go while carrying a glass of water
TUG-O: Timed-up and go with an obstacle to step over
UDysRS: Unified dyskinesia rating scale
UPDRS: Unified Parkinson's disease rating scale.
